# Clinical value and risk factors of compression supporting screwsin patients with femoral neck fractures

**DOI:** 10.1186/s13018-024-04873-y

**Published:** 2024-09-19

**Authors:** Yazhou Zhang, Guanqing Li, Zhi Tian, Can Cao, Changmao Qiu, Xicheng Li

**Affiliations:** 1 Department of Orthopedics, Hebei General Hospital, No. 348 Heping East Road, Shijiazhuang, 050051 Hebei China; 2 Department of Emergency, Hebei General Hospital, Shijiazhuang, 050051 Hebei China

**Keywords:** Compression supporting screw, Femoral neck fracture, Application value, Risk factors

## Abstract

**Background:**

This study was performed with attempt to explore the clinical value and risk factors of compression supporting screws for the treatment of femoral neck fractures.

**Methods:**

This retrospective analysis enrolled 102 patients with femoral neck fractures who admitted to our hospital from June 2020 to June 2022. Based on different screws during the operation, the participants were allocated into hollow screw group (52 cases, conventional fixation of parallel partial-thread hollow screw) and compression screw group (50 cases, compression screw fixation).

**Result:**

The incidence of complications (including internal fixation failure, nonunion, a vascular necrosis of the femoral head, shortening of the femoral neck by less than 10 mm, and lateral screw withdrawal, of the affected limb) in the compression screw group were significantly lower than those in the hollow screw group (*P* < 0.05). Patients enrolled in this study were followed up for 9 to 14 months, with an average follow-up time of (12.09 ± 1.87) months.The pain degree at 3 days, 10 days, and the last follow-up after operation in the compression screw group was evidently lower than that in the hollow screw group (*P* < 0.05). At the last follow-up, the improvment in hip joint function was more significant in the compression screw group than in the hollow screw group (*P* < 0.05). Univariate logistic regression analysis showed that the risk factors for complications in the treatment of femoral neck fractures with compression supporting screws were age, Pauwels type III fracture (modified Pauwels classification), and hip joint (≥ 90 points). In addition, the result of multivariate logistic regression analysis showed that the risk factors for complications in the treatment of femoral neck fractures with compression supporting screws were age, Pauwels type III fracture (modified Pauwels classification).

**Conclusion:**

Our findings demonstrated beneficial outcomes obtained by using compression supporting screw, in terms of effectively enhancing the recovery of patients with femoral neck fracture and reducing the associated complications.

## Introduction

Femoral neck fractures are a prevalent condition in orthopedics, with a high incidence rate due to factors such as increasing traffic accidents and an aging population in recent years [1]. Relevant data [2] have reported a high incidence rate caused by femoral neck fractures: 3.6% of total body fractures and 48–54% of hip fractures. In elderly patients with poor bone mass, femoral neck fractures were mostly caused by low energy injury. In young patients, it is mostly caused by high-energy injuries, such as falling from high places, car accidents and so on. A recent study in China indicated that by 2025, the number of femoral neck fractures and the economic cost of the treatment will increase significantly, which will put a heavy burden on society and families [3]. With the development of industry, transportation and urbanization in China, middle-aged and young patients with femoral neck fracture caused by high energy injury will also be more common. Because of the complex biomechanics of the femoral head and neck and the special relationship of local blood supply and anatomy, the fracture of the femoral neck is often associated with high risk of complications such as bone nonunion and femoral head necrosis. According to literature statistics [4, 5], the rate of bone nonunion after femoral neck fracture can reach 8−39%, and the rate of femoral head ischemic necrosis can reach 14.3–28%. In addition, other complications include internal fixation failure, femoral neck shortening and varus. These poor outcomes can lead to joint pain or limb dysfunction that will seriously affect the patient’s quality of life. Therefore, it is of great significance to rationally select the treatment of femoral neck fracture and improve the prognosis of patients. Since femoral neck fractures are mostly caused by high-energy injury, accompanied by comminution and displacement of the fracture site, the biological environment for its healing is relatively unstable, along with compromised blood flow and biomechanics in the fracture broken end and the femoral head. These factors can lead to increased complications of high-energy injury such as internal fixation failure, nonunion, and avascular necrosis of the femoral head post-fracture operation. However, controversy exists with regard to the optimal approach to treating femoral neck fracture, and issues such as shortening of the femoral neck during the bone remodeling process are also a concern [6]. Currently, the main goal of surgical treatment of femoral neck fracture is to retain the femoral head as much as possible, pressurize the fracture end, and achieve anatomical reduction to resist the rotational stress and shear stress [7]. Therefore, it is particularly important to select appropriate and stable internal fixation. In clinical practice, several internal fixation methods exist for the treatment of femoral neck fractures. Among these, partially threaded hollow screws remains a crucial option, offering benefits such as shorter operation time, minimal trauma, and reduced intraoperative bleeding [8]. With technological advancements, the compression achieved by three parallel hollow screws at the fracture site can provide initial stability, allowing the fracture block to slide along the screw insertion direction under patient load for secondary stability [8]. However, the utilization of partially threaded hollow screws often leads to internal fixation failures and subsequent femoral neck shortening after fixation [9]. In recent years, the full-thread headless hollow screw, referred to as compression supporting screw, has gained prominence in the treatment of fracture patients. Previous studies have showed that, compared to partially threaded hollow screws, compression supporting screws can reduce the occurrence of femoral neck shortening. Another study [10] also depicted that the use of compression supporting screws may reduce the occurrence of bone nonunion and internal fixation failure. However, there is limited research on whether screw compression can improve the prognosis of patients with femoral neck fracture. Also, the application value and risk factors of compression support screws in patients with femoral neck fracture have not been analyzed in previous studies. Herein, we aimed to analyze the application value and risk factor associated with the use of compression supporting screws in patients with femoral neck fractures.

## Participants and methods

### Participants

**Fig. 1:**
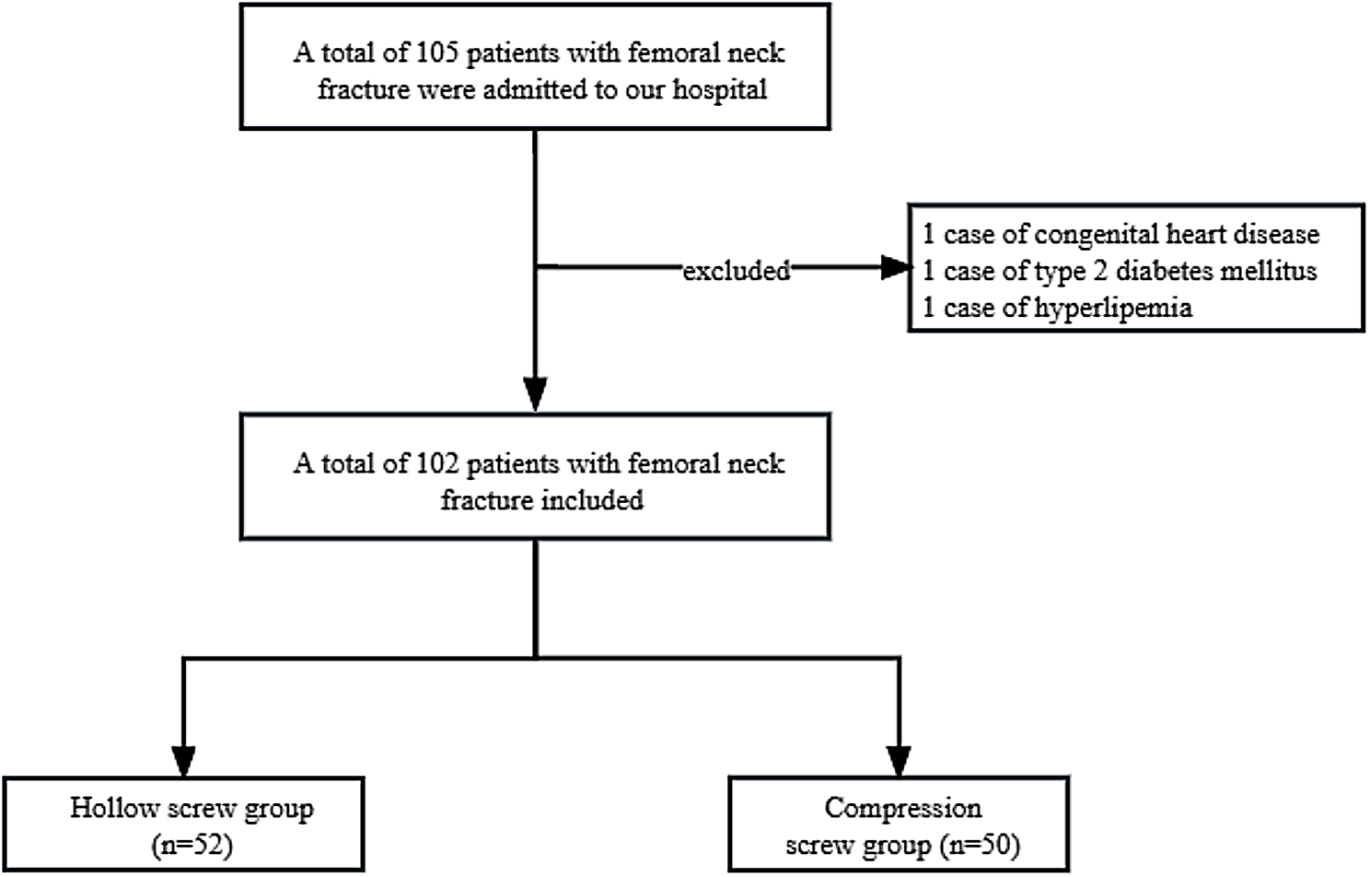
Patient inclusion flow chart

This retrospective analysis involved 102 patients with femoral neck fractures admitted to our hospital from June 2020 to June 2022. All patients were admitted within 6 h of injury and underwent surgery within 48 h. Based on different screws during operation, the participants were allocated into the hollow screw group (52 cases, conventional fixation of parallel partial-thread hollow screw) and the compression screw group (50 cases, compression screw fixation) (The flow chart is shown in Fig. 1).

### Selection criteria

Inclusion criteria: (1) Patients with 3-I B-type femoral neck fractures confirmed by X-ray or CT scan; (2) Absence of heart, brain, lung, kidney, or other major organ diseases; (3) Patients with closed femoral neck fractures; (4) Patients underwent surgery within 2 days after injury; (5) Treated with conventional parallel partial thread hollow screw fixation or compression screw fixation treatment.

Exclusion criteria: (1) Patients with concomitant fractures of the ipsilateral lower limb, femoral head, pelvis, or acetabulum; (2) Patients with multiple fractures (injury severity score > 16 points); (3) Patients who underwent open reduction and internal fixation, with a history of surgical procedures, deformities, developmental abnormalities of the ipsilateral hip joint or femur, orpathological or old fractures; (4) Patients lost to follow-up or those who refused to participate in this study; (5) Patients with risk factors for femoral head necrosis, such as long-term heavy alcohol consumption, or use of steroid drugs, etc.; (6) Patients with abnormal coagulation function; (7) Patients with pre-existing limb dysfunction impeding their ability to perform activities of daily living; (8) Patients with pelvic fractures or other lower limb fractures; (9) Patients with hip arthritis, femoral head ischemic necrosis, or other conditions affecting hip joint function.

This research protocol complied with the relevant requirements of the Helsinki Declaration of the World Medical Association.The Ethical Committee for Hebei General Hospital reviewed and approved the study protocol. All participants were aware of this study and signed the informed consents.

### Methods

#### Reduction methods

All surgeries in this study were performed by the same orthopedic specialist with over 15 years of experience. After entering the operating room, the affected limb of patient was placed on the traction bed. The length of the limb and the axial displacement of the fracture were restored by longitudinal traction with the help of the C-arm fluoroscopy machine, and the rotation deformity of the fracture was corrected by internal and external rotation of the lower limb. The acceptable reduction criteria for intraoperative judgment was that the hip joint was slightly everted or reached anatomical reduction (femoral neck trunk angle 130°-150°) in the positive perspective, and there was no anteroposterior tilt of the femoral head (anteversion angle ≤ 15°) in the lateral perspective. If the reduction was not satisfactory, such as a forward or backward displacement of the proximal end of the fracture, the reduction downward could be performed by inserting a mandril through a small incision at the fracture end of the femoral shaft.

#### Compression screw group

After the reduction, the guide needle was inserted into the subchondral bone of the femoral head, and the depth was measured and the cortex was opened. Subsequently, one compression screw (Asnis III, Stryker) at the proximal end and two full-thread headless hollow screws at the distal end were inserted in a parallel “inverted triangle” layout–mixed screw type. When placing a partial-thread hollow screw in the middle of the proximal femoral neck, the fracture section could be moderately compressed to close the fracture space. And then a compression screw was placed in the anterior lower quadrant to maintain the length of the neck. Before each compression screw was inserted, it was necessary to tap the lateral cortex of the proximal femur along the guide to reduce the torque. Meanwhile, performer should ensure that the compression screw tail was cut and locked with the cortex of the proximal femur to prevent the screw from being exposed outside the cortex for too long.

In the control group, three 6.5 mm partially threaded hollow screws were routinely inserted according to the standard technique.

#### Postoperative follow-up

All affected limbs were routinely given cefuroxime for infection prevention, tranexamic acid for hemostasis, and parecoxib for pain relief. Amicar was administered to control bleeding in patients based on the intraoperative and postoperative bleeding situation. Routine anticoagulation treatment was started 8 h after operation. After awakening from anesthesia, the patients were guided to perform the ankle pump movement and isometric contraction training of quadriceps femoris. After extubating, researchers reviewed the X-ray film, and guided the patient to use the walking aid to get out of bed as soon as possible after confirming the position of the prosthesis was satisfactory.

Each patient received follow-up evaluations three times. The initial follow-up occurred at 3 months postoperatively, during which pain severity and hip joint function were assessed. If the pain severity score was below 2 points and the hip joint function score was greater than 75 points, no X-ray examination was necessary. The second follow-up took place at 6 months postoperatively, during which pain severity and hip joint function were reassessed, and an X-ray examination was offered based on patient preference. The final follow-up occurred at 9 months postoperatively, during which pain severity and hip joint function were reassessed.

### Observation indexes

The operation time, intraoperative bleeding, hospital stay, fracture reduction quality and operation failure rate were recorded. The fracture healing time and complications of the affected limbs in the two groups were counted. The hip joint function of the two groups was compared before operation and at the last follow-up. We also analyzed the pain degree of the two groups at different times. Univariate/multivariate logistic regression analyses were applied to analyze the risk factors of complications in patients with femoral neck fractures treated with compression supporting screws.

#### Fracture reduction quality

According to the degree of fracture displacement and angulation, fracture reduction quality was determined [11]: (1) Excellent: fracture displacement < 2 mm or fracture angulation < 5° in two fluoroscopy planes; (2) Good: displacement 2–5 mm or angulation 5° − 10 °; (3) Moderate: displacement 6–10 mm or angulation 11° − 20°; (4) Poor: displacement > 10 mm or angulation in any plane > 20 °.

#### Pain degree

The pain visual analogue scale (VAS) [12] was used to assess the pain degree before operation, 3 days after operation, 10 days after operation and at the last follow-up, with a score of 0–10 points. The higher the score, the more obvious the pain degree of the patient.

#### Hip joint function

We evaluated the hip joint function before operation and at the last follow-up based on the Harris Hip Score (HHS) [13]. The HHS has four grades: excellent (90–100), good (80–89), fair (70–79), and poor (< 70). Excellent rate=(excellent + good)/total number of cases × 100%.

### Statistical analysis

SPSS 21.0 software was used to analyze the data. The normality test was conducted for continuous variables. The operation time, intraoperative bleeding, hospital stay, and other measurement data were expressed as mean ± standard deviation (SD). For variables that satisfied normal distribution, the Student t-test was employed for statistical comparison. The counting data was expressed in percentage (%), using chi-square test. Univariate/multivariate logistic regression analyses were used to analyze the risk factors of complications in patients harboring femoral neck fractures treated with compression supporting screws. A *P* < 0.05 was considered statistically significant.

## Results

### Comparison of baseline characteristics

In hollow screw group, there were 32 males and 20 females, aged from 29 to 74 years, with an average age of (50.09 ± 17.19) years; body mass index (BMI): 19–25 kg/m^2^, averaged BMI (22.73 ± 2.07) kg/m^2^. In compression screw group, there were 29 males and 21 females, aged from 28 to 73 years, with an average age of (50.03 ± 17.23) years; BMI: 19–25 kg/m^2^, averaged BMI (22.67 ± 2.11) kg/m^2^. The baseline characteristics between the two groups were comparable (*P* > 0.05), as shown in Table 1.

**Table 1:** Comparison of general data

Index	Compression screw group (*n* = 50)	Hollow screw group (*n* = 52)	χ^2^/t	*P* value
Age (year)	50.09 ± 17.19	50.03 ± 17.23	0.018	0.986
BMI (kg/m^2^)	22.73 ± 2.07	22.67 ± 2.11	0.145	0.885
Sex (male/female)			0.133	0.716
Male	29 (58.0%)	32 (61.5%)		
Female	21 (42.0%)	20 (38.5%)		
Side of injury (n)			0.044	0.834
Left	23 (46.0%)	25 (48.1%)		
Right	27 (54.0%)	27 (51.9%)		
Cause of injury (n)			0.348	0.951
Traffic injury	25 (50.0%)	23 (44.2%)		
Falling injury	12 (24.0%)	14 (26.9%)		
Fall and hurt oneself	8 (16.0%)	9 (17.3%)		
Sports injury	5 (10.0%)	6 (11.6%)		
Modified pauwels classification (n)			0.129	0.719
Type I-II	20 (40.0%)	19 (36.5%)		
Type III	30 (60.0%)	33 (63.5%)		
Smoking (n)	12 (24.0%)	15 (28.8%)	0.308	0.59
Drinking (n)	9 (18.0%)	14 (26.9%)	1.162	0.281
Comorbid diabetes	17 (34.0%)	19 (36.5%)	0.072	0.789

### Comparison of operation time, intraoperative bleeding, hospital stay, fracture reduction quality and complications

There were no significant differences between the two groups in operation time, intraoperative bleeding, hospital stay, and fracture reduction quality (*P* > 0.05). The complications, including internal fixation failure, nonunion, shortening of the femoral neck by less than 10 mm, and lateral screw withdrawal, of the affected limb in the compression screw group were significantly lower than those in the hollow screw group (*P* < 0.05), as laid out in Table 2.

**Table 2:** Comparison of operation time, intraoperative bleeding, hospital stay, fracture reduction quality, fracture healing time based on imaging evaluation and complications

Index	Compression screw group (*n* = 50)	Hollow screw group (*n* = 52)	χ^2^/t	*P* value
Operation time (min)	45.12 ± 10.23	47.87 ± 10.92	-1.311	0.193
Intraoperative bleeding (mL)	75.82 ± 18.71	80.48 ± 17.92	-1.285	0.202
Hospital stay (d)	9.76 ± 2.87	10.09 ± 2.76	-0.592	0.555
Fracture reduction quality (n)			0.488	0.181
Excellent	45 (90.0%)	38 (73.1%)		
Good	3 (6.0%)	9 (17.3%)		
Moderate	1 (2.0%)	3 (5.7%)		
Poor	1 (2.0%)	2 (3.9%)		
Complications (n)				
Internal fixation failure	1 (2.0%)	7 (13.5%)	4.633	< 0.001
Nonunion	1 (2.0%)	8 (15.4%)	5.676	0.017
Avascular necrosis of the femoral head	1 (2.0%)	3 (5.8%)	0.961	0.327
< 10 mm femoral neck shortening	2 (4.0%)	16 (30.8%)	12.569	< 0.001
Outward screw withdrawal	4 (8.0%)	23 (44.2%)	17.191	< 0.001
Inward displacement	0 (0)	0 (0)	0	0

### Comparison of hip joint function and pain degree

**Fig. 2:**
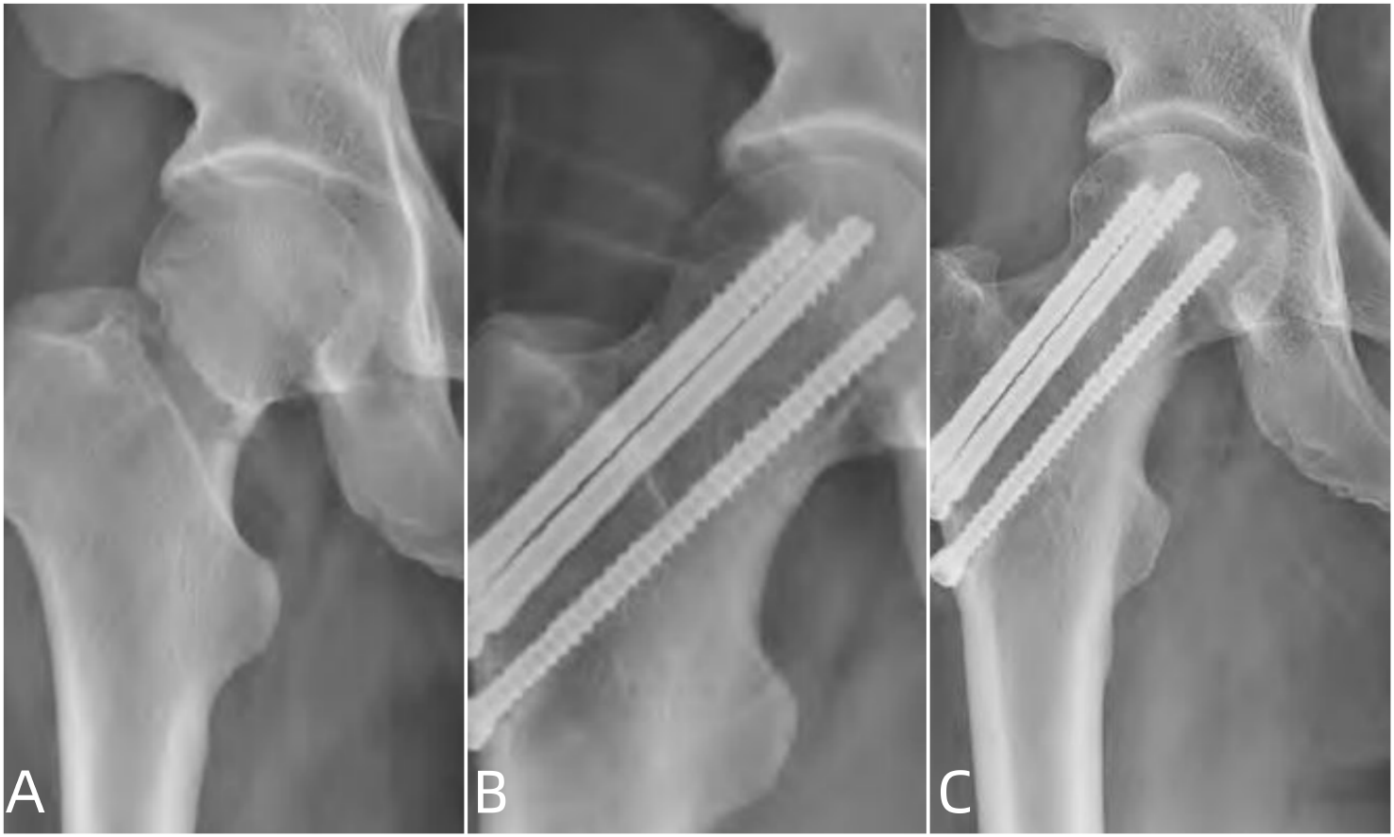
A 58-year-old type B femoral neck fracture (Pauwels II) female patient treated with compression screw. **A** shows the anteroposterior position before the operation, while **B** shows the postoperative anteroposterior position 1 week after operation, indicating that the fracture was in good position for reduction and internal fixation, and **C** shows the anteriorposterior position 12 months after surgery, showing good fracture healing without screw withdrawal, femoral neck shortening, or avascular necrosis of the femoral head

**Fig. 3:**
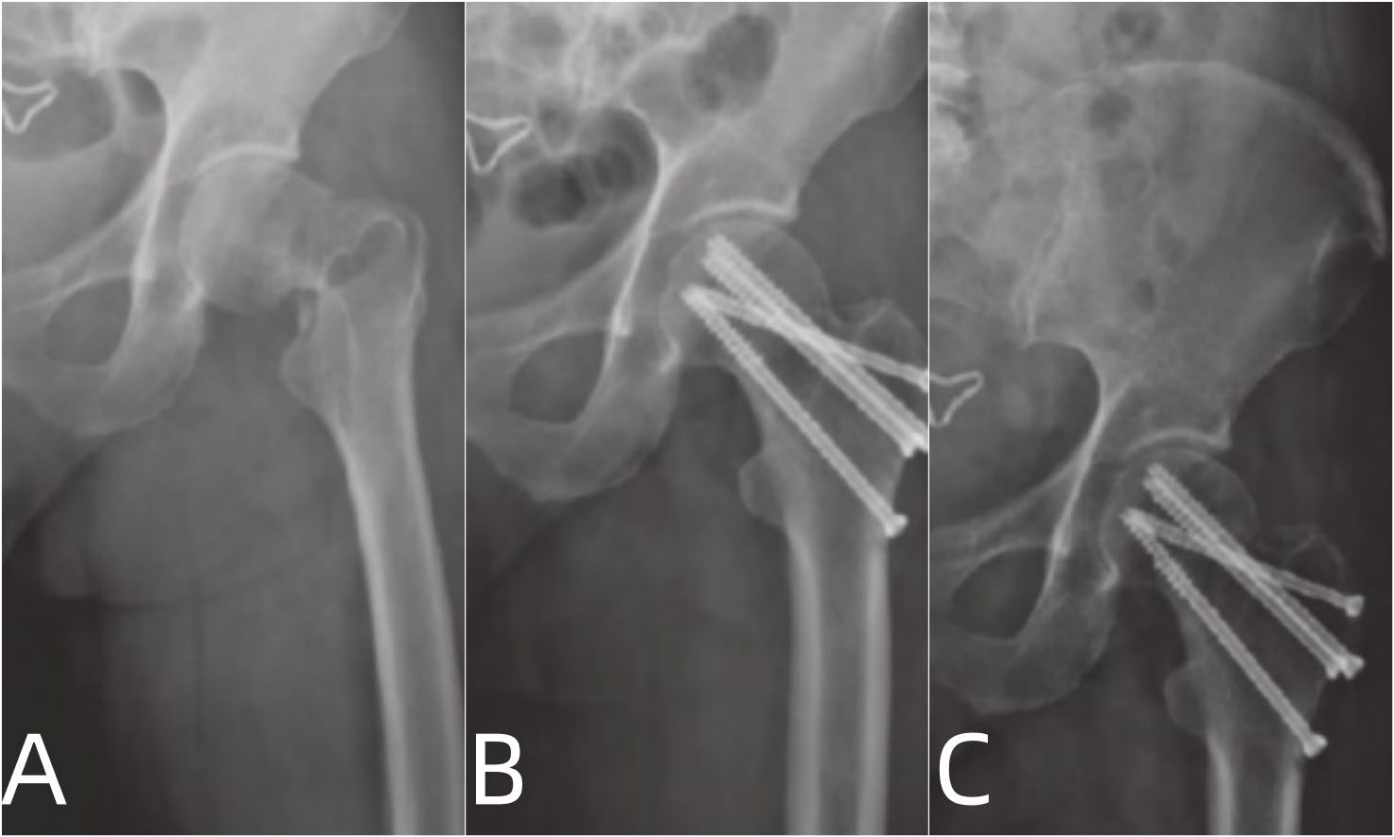
Anteroposterior radiographs of a 37-year-old male patient with a left femoral neck fracture (Pauwels type II, Garden subtype). **A** shows the radiograph before the operation, **B** shows the radiograph 1 week after surgery indicating that the fracture was in a good position for reduction and internal fixation, and **C** exhibits radiograph 6 months after surgery showing a reasonable prognosis

The patients enrolled in this study were followed up for 9 to 14 months, with an average follow-up time of (12.09 ± 1.87) months. There was no significant difference in the hip joint function between the two groups before operation (*P* > 0.05). However, the pain degree at 3 days, 10 days, and the last follow-up after operation in the compression screw group was evidently lower than that in the hollow screw group (*P* < 0.05). The hip joint function at the last follow-up of the compression screw group improved more than that of the hollow screw group (*P* < 0.05) (Table 3; Figs. 2 and 3).

**Table 3:** Comparison of hip joint function and pain degree

Index	Time	Compression screw group (*n* = 50)	Hollow screw group (*n* = 52)	t	*P*
Hip joint (point)	Before operation	40.87 ± 10.39	41.91 ± 10.45	-0.543	0.588
	Last follow-up	90.29 ± 10.01	83.91 ± 10.32	3.168	0.002
Pain degree (point)	Before operation	7.87 ± 1.09	7.76 ± 1.04	0.522	0.603
	3d after operation	7.05 ± 1.23	7.01 ± 1.32	0.158	0.875
	10d after operation	1.67 ± 0.46	1.92 ± 0.52	-2.568	0.012
	Last follow-up	1.02 ± 0.23	1.45 ± 0.27	-8.643	< 0.001

### Univariate logistic regression analysis of risk factors for complications in patients with femoral neck fractures treated with compression supporting screws

The independent variables were set as general data, operation time, intraoperative bleeding, hospital stay, fracture reduction quality, hip joint function and pain degree. The dependent variables were the complications of patients with femoral neck fracture treated with compression supporting screw. The univariate logistic regression analysis showed that, the risk factors for complications of patients with femoral neck fracture treated with compression supporting screw were age and hip joint (≥ 90 points) (Table 4).

**Table 4:** Univariate logistic regression analysis of risk factors for complications in patients with femoral neck fractures treated with compression supporting screws

Index	β	SE	Wdlodχ^2^ value	OR(95%CI)	*P* value
Duration of surgery (> 45 min)	0.945	0.327	6.981	2.461(1.317–3.871)	0.003
Intraoperative bleeding (> 75 ml)	0.387	0.182	4.281	1.409(1.021–2.516)	0.029
Hospital stay (≥ 10d)	0.373	0.792	0.206	1.362(0.371–6.381)	0.638
Fracture reduction quality (poor)	0.789	0.652	1.459	2.209(0.608–4.761)	0.263
Hip joint (≥ 90 points)	0.932	0.317	8.913	2.371(1.187–4.076)	0.003
Pain degree (≥ 2 points)	0.419	0.287	2.176	1.521(0.865–2.891)	0.209

### Multivariate logistic regression analysis of risk factors for complications in patients with femoral neck fractures treated with compression supporting screws

The independent variables included age and hip joint score (≥ 90 points).The dependent variables were the occurrence of complications in patients with femoral neck fractures treated with compression supporting screws. The results showed that age is significant risk factor for complications (Table 5).

**Table 5:** Multivariate logistic regression analysis of risk factors for complications in patients with femoral neck fractures treated with compression supporting screws

Index	β	SE	Wdlodχ^2^ value	OR(95%CI)	*P* value
Duration of surgery (> 45 min)	0.945	0.327	6.981	2.461(1.317–3.871)	0.003
Intraoperative bleeding (> 75 ml)	0.387	0.182	4.281	1.409(1.021–2.516)	0.029
Hospital stay (≥ 10d)	0.373	0.792	0.206	1.362(0.371–6.381)	0.638
Fracture reduction quality (poor)	0.789	0.652	1.459	2.209(0.608–4.761)	0.263
Hip joint (≥ 90 points)	0.932	0.317	8.913	2.371(1.187–4.076)	0.003
Pain degree (≥ 2 points)	0.419	0.287	2.176	1.521(0.865–2.891)	0.209

## Discussion

Femoral neck fractures are a common type of hip fracture, and the treatment may lead to complications such as nonunion and avascular necrosis of the femoral head, which significantly impacting patients’ quality of life [14] . Various internal fixation methods, including full-thread screws, partial-thread hollow screws, dynamic hip screws, and locking plates, are currently common applied in clinical practice. However, selecting the optimal internal fixation method remains challenging [15–17]. In the context of internal fixation of femoral neck fractures, two types of full-thread screws are common: the full-thread hollow screw with a standard head, cylindrical body, and equal pitch, and the compression supporting screw with thread head, conical body, and gradual pitch. The compression supporting screw combines characteristics of both the full-thread hollow screw and the partial-thread hollow screw. It offers static sliding or compression (slow and progressive sliding) and provides length stability due to its length control structure. The compression supporting screw achieves compression or sliding of the two fracture blocks via two potential mechanisms: firstly, the full-thread taper design assists in anchoring the screw threads into new bone with each movement forward, thus attaining compression and maximum pullout strength between the fracture ends; secondly, the variable pitch of the screw thread facilitates rapid screw insertion and gradual pressure exertion on the two fracture blocks. Moreover, the layout of the full-thread is effective in managing the cyclic load applied by the body during fracture healing and contributes to stabilizing the length of the full-thread hollow screw [18]. In addition, pressure screws and full-threaded screws are length stabilized internal fixations. Both the head and tail of the pressure screw are threaded. The screw tail thread is conical in design, and its thread distance is smaller and the diameter is larger than that of the screw head. This design feature enables it to exert strong pressure on the fracture end of the femoral neck, providing initial stable conditions for fracture healing. Because the thread of the tail is driven into the lateral cortex of the proximal femur, the holding force is stronger than that of the single hollow screw, which can effectively reduce the incidence of screw withdrawal and femoral neck shortening. The full-thread screw is designed with equal pitch, which can exert static pressure on the fracture end. It has good supporting ability of the fracture end, but the pressurizing force is insufficient. The combination of pressure screw and full thread screw can exert the compound effect of double-end pressure and medial support to achieve firm pressure at the fold end and provide strong support and fixation for fracture healing [18].

Chen Tao et al. [19] showed that the use of hollow compression screws could shorten the operation time, reduce the amount of bleeding, and accelerate the bone healing. According to Zhang et al. [20], the treatment of vertical femoral neck with compression internal supporting screw technology could reduce the incidence of nonunion, internal fixation and deformity of femoral neck varus. Moreover, Zhang et al. [18] depicted that compared to conventional partially threaded hollow screws, compression supporting screws showed certain advantages in fixing femoral neck fractures, especially in high-energy vertical unstable fractures. Similar with our results, patients with femoral neck fractures treated with compression supporting screws obtained similar operation time, intraoperative bleeding, hospital stay, and fracture reduction quality as hollow screws, while it was able to reduce the occurrence of complications such as internal fixation failure, nonunion, avascular necrosis of the femoral head, shortening of the femoral neck by less than 10 mm, and lateral screw withdrawal. Okcu et al. [21] reported that patients with femoral neck fractures undergoing compression supporting screw internal fixation exhibited shorter fracture healing time and lower complication rate compared to as those treated with hollow screws. However, another study [22] pointed out that, the incidence of varus collapse and femoral neck shortening revealed no decrease in patients with femoral neck fractures treated with compression supporting screw internal fixation compared to those treated with hollow screws, with no significant difference (*P* > 0.05). But it is important to note that the main participants of the above mentioned study were elderly patients, and the main cause of femoral neck fracture was low-energy injury (Pauwels I and II fractures accounted for 90%). Weil et al. [23] compared three fully threaded screws and three partially threaded cannulated screws for internal fixation of femoral neck fractures. Two years after the operation, it was found that there was no significant difference in the average length of femoral neck shortening between the two methods, and there was no significant difference in the incidence of complications such as varus deformity, osteonecrosis and nonunion. The study of Parker et al. [24] pointed out that there was no significant difference in the risk of complications such as osteonecrosis, nonunion and re-operation after the treatment with full thread screw and partial thread screw, indicating that the effect of screw thread length on the treatment of femoral neck fracture may not be certain. In this study, the patients with femoral neck fractures were mainly middle-aged and elderly, with more Pauwels III fracture than those with Pauwels I; and II fractures, hence the different results. In addition, 1 case of internal fixation failure occurred in the treatment of femoral neck fractures with compression supporting screws in this study. The reason may attribute to the large angle of femoral neck retroversion angle (> 20°).

Jin et al. [25] found that hollow pressure screw fixation in TF patients of different ages has a definite effect, and does not affect the recovery of hip function or increase the occurrence of related complications. Zhang Zhiwen et al. [26]. The operative time required for hollow nail internal fixation for the treatment of femoral neck fracture of Pauwels type III in young adults was (47.83 ± 5.12) min. Qian Jianrong et al. [27]. The operative time of hollow nail internal fixation for senile Garden I and II femoral neck fractures was (49.6 ± 14.8) min.The results of this study suggest that both methods can reduce the surgery time, length of hospital stay, intraoperative blood loss, and complications, while improving the quality of fracture reduction and expediting imaging-based healing of the affected limb. However, it was observed that patients over 60 years of age exhibited prolonged hospital stays and imaging-based fracture healing times compared to those under 60 years of age. Additionally, the order cohort displayed lower fracture reduction quality in comparison to the younger group.

The Harris hip score is widely utilized to comprehensively evaluate hip joint function in clinical practice[28]. Zhao et al. [28] proposed that utilizing compression screws for treating femoral neck fractures could improve the Harris score and expedite fracture healing. The results of this study revealed that, at 3 days, 10 days after surgery, and during the final follow-up, pain levels among patients over 60 years old in both groups were lower than those in the hollow screw group, and their hip joint function was superior to that of individuals under 60 years old (*P* > 0.05).

Xiao et al. [29] reported that the treatment of femoral neck fractures with hollow compression screws, involving the insertion of a sartorius muscle iliac bone flap, promoted femoral neck fracture healing, improved hip joint function recovery, and reduced the occurrence of related complications. According to Weil et al. [23], the use of fully threaded screws can significantly reduce femoral neck shortening after internal fixation without increasing the incidence of bone nonunion and osteonecrosis, which is conducive to improving the treatment effect of femoral neck fracture. Compatible with our results, it suggested that employing with compression support screws for femoral neck fracture treatment yielded beneficial outcomes in restoring the function of hip joint, alleviating pain, and promoting patient recovery.

According to Tan et al. [30], age is a significant risk factor for postoperative complications of femoral neck fractures. Hou et al. [31] emphasized that the occurrence of complications in patients with femoral neck fractures after internal fixation treatment was closely related to fracture classification. Wang et al. [32] also pointed out that age is the risk factor for complications after the operation of femoral neck fractures. These findings are compatible with the univariate logistic regression analyses conducted in our study, which identified age is risk factor for complications in patients with femoral neck fractures treated with compression supporting screws. Based on multivariate logistic regression analysis, the risk factor for complications in patients treated with compression supporting screws is age, indicating age is risk factors for complications in patients with femoral neck fractures treated with compression supporting screws.

In conclusion, patients with femoral neck fractures treated with compression supporting screws exhibit comparable operation time, intraoperative bleeding, hospital stay, and fracture reduction quality as those treated with hollow screws. However, the use of compression supporting screws results in superior outcomes, including reduced occurrence of complications such as internal fixation failure, nonunion, avascular necrosis of the femoral head, shortening of the femoral neck by less than 10 mm, and lateral screw withdrawal. This approach proves beneficial in restoring hip joint function, alleviating pain, and promoting recovery. Furthermore, our study identifies age as risk factor for complications in patients with femoral neck fractures treated with compression supporting screws. Admittedly, there are several limitations that should be acknowledged. Considering the short follow-up time and retrospective nature of our study, other factors may affect the difference of postoperative complications between the two groups. Hence, further RCTs with longer following-ups are warranted.

## Data Availability

All data generated or analyzed during this study are included in this published article.
